# Determination of risk factors for acute renal failure after cardiac surgery

**DOI:** 10.1186/2197-425X-3-S1-A634

**Published:** 2015-10-01

**Authors:** TA Ayazoğlu, A Baysal, O Akman, A Özensoy

**Affiliations:** Anesthesiology and Reanimation, Medeniyet University Goztepe Research and Training Hospital, Istanbul, Turkey; Anesthesiology and Reanimation, Kartal Kosuyolu Research and Training Hospital, Istanbul, Turkey; Anesthesiology and Reanimation, Via Hospital Sancaktepe, Istanbul, Turkey

## Introduction

It is vital to determine the risk factors related to acute kidney injury (AKI). The modified RIFLE (risk, injury, failure, loss of kidney function, and end-stage renal failure) classification based on changes in serum creatinine (sCr) relative to the baseline condition may provide valuable data to determine patients in need for renal replacement therapy (RRT) ([1]).

## Objectives

Our aim was to assess perioperative risk factors for AKI for cardiac surgery.

## Methods

We prospectively collected data of 1750 consecutive cardiac surgery patients. AKI was defined by RIFLE system and a modified criteria was included to determine patients with acute need for RRT in the failure class. Risk factors including pre-operative, operative and post-operative variables were investigated by univariate and multivariate analysis.

## Results

The incidence of AKI was 15.3% (n = 267) and of these RRT was performed on 69.6 % (n=185). The mortality rate among patients with AKI was 54 (20%) of 267 patients. Preoperative factors causing AKI revealed; angiotensin converting enzyme (ACE) inhibitors, prior recent acute myocardial infact (AMI) (< 6 weeks), euroscore. Postoperative factors causing AKI revealed; cardiopulmonary bypass (CPB) time, need for longer duration of vasoactive drugs and a higher arterial lactate level within 24 hours in ICU (p < 0.05). Multiple logistic regression analysis revealed; AMI (hazard ratio (HR)= 1.08, 95% confidence interval (CI) 1.01 to 1.15, p = 0.03), CPB time (hazard ratio (HR) = 1.81, 95% confidence interval (CI) 1.28 to 2.56, p = 0.001), and postoperative lactate level on day 1 (hazard ratio (HR) = 10.57, 95% confidence interval (CI) 1.90 to 58.81, p = 0.007), as independent risk factors for RRT. The worst outcomes, including in-hospital mortality, were associated with the worst RIFLE class (p < 0.001).

## Conclusions

In development of AKI, several variables may play role. Our study demonstrated that preoperative recent AMI, CPB time and postoperative lactate level on day 1 are independent risk factors for RRT after cardiac surgery.
